# Proteome Analysis of the Hypothalamic Arcuate Nucleus in Chronic High-Fat Diet-Induced Obesity

**DOI:** 10.1155/2021/3501770

**Published:** 2021-11-18

**Authors:** Chang Yeon Kim, Jang Ho Ahn, Do Hyun Han, Cherl NamKoong, Hyung Jin Choi

**Affiliations:** ^1^Functional Neuroanatomy of Metabolism Regulation Laboratory, Department of Anatomy and Cell Biology, Department of Biomedical Sciences, Seoul National University College of Medicine, Seoul, Republic of Korea; ^2^Proteomics Core Facility, Biomedical Research Institute, Seoul National University Hospital, Seoul, Republic of Korea; ^3^Neuroscience Research Institute, Seoul National University College of Medicine, Seoul, Republic of Korea; ^4^Wide River Institute of Immunology, Seoul National University, Hong Cheon, Republic of Korea

## Abstract

The hypothalamus plays a central role in the integrated regulation of feeding and energy homeostasis. The hypothalamic arcuate nucleus (ARC) contains a population of neurons that express orexigenic and anorexigenic factors and is thought to control feeding behavior via several neuronal circuits. In this study, a comparative proteomic analysis of low-fat control diet- (LFD-) and high-fat diet- (HFD-) induced hypothalamic ARC was performed to identify differentially expressed proteins (DEPs) related to changes in body weight. In the ARC in the hypothalamus, 6621 proteins (FDR < 0.01) were detected, and 178 proteins were categorized as DEPs (89 upregulated and 89 downregulated in the HFD group). Among the Gene Ontology molecular function terms associated with the DEPs, protein binding was the most significant. Fibroblast growth factor receptor substrate 2 (Frs2) and SHC adaptor protein 3 (Shc3) were related to protein binding and involved in the neurotrophin signaling pathway according to Kyoto Encyclopedia of Genes and Genomes analysis. Furthermore, high-precision quantitative proteomic analysis revealed that the protein profile of the ARC in mice with HFD-induced obesity differed from that in LFD mice, thereby offering insight into the molecular basis of feeding regulation and suggesting Frs2 and Shc3 as novel treatment targets for central anorexigenic signal induction.

## 1. Introduction

Obesity is a common issue worldwide and a cause of various disorders, including hypertension, diabetes, and cardiovascular disorders [1]. Several neural circuits in the brain, including the hypothalamus, solitary nucleus, midbrain, and limbic system, are involved in the maintenance of body weight and appetite [2]. Leptin and insulin regulate feeding behavior and energy homeostasis in the hypothalamus [3, 4], and the arcuate nucleus (ARC) of the hypothalamus is involved in feeding and energy expenditure [5].

The ARC is anatomically located at the lateral wall of the third ventricle and lies directly above the pituitary gland upon the median eminence in the hypothalamus. Several types of neurons in the feeding circuit of the ARC have been discovered, including those expressing agouti-related protein/neuropeptide Y (agouti-related protein [AgRP]/NPY) or proopiomelanocortin (POMC) derivatives [6, 7]. Orexigenic AgRP neurons are negatively regulated by adipocyte-derived leptin, whereas anorexigenic POMC neurons are triggered by leptin to release mainly *α*-MSH derivatives that affect nuclei, including the paraventricular nucleus (PVN) of the brain [8, 9]. Aside from several peptides and neural pathways, the molecular mechanisms underlying the regulation of the feeding circuit in the ARC have not been fully elucidated. Considering its pivotal role in food intake and satiety, we hypothesized that proteome profiling of the ARC would uncover critical circuits related to obesity. Although proteomic analyses of the hypothalamus in a high-fat diet- (HFD-) induced obesity (DIO) model have been performed [9], few studies have focused specifically on the ARC.

In this study, we characterized the ARC proteome in order to identify protein alterations in mice exposed to an HFD and provide new insights into the molecular basis of appetite regulation and energy metabolism.

## 2. Materials and Methods

### 2.1. Animals

Male C57BL/6 mice (7 weeks old; 20–25 g; *n* = 10) were purchased from Orient Bio (Seongnam, Korea) and maintained at 23 ± 1°C under a 12 h light/dark cycle starting at 8:00 A.M., with *ad libitum* access to the designated type of food and water in a breeding room. Body weights were measured weekly.

### 2.2. Ethics Statement

This study was carried out in accordance with the Guide for the Care and Use of Laboratory Animals of Seoul National University Hospital. The protocol was approved by the Institutional Animal Care and Use Committee of the Seoul National University Hospital (protocol 18-0154-S1A1).

### 2.3. DIO Model

All mice underwent a week of adjustment to the housing environment and were fed a chow diet. The mice were then randomly assigned to two groups (*n* = 5/group) for the 8-week experimental period. One group was fed a low-fat control diet (LFD; rodent diet with 10% of energy from fat and 3.8 kcal/g), and the other was fed an HFD (60% of energy from fat and 5.2 kcal/g) *ad libitum* for 8 weeks (D12450B and D12492, respectively; Research Diets, Brunswick, NJ, USA). Mean body weights were measured weekly in both groups to evaluate the reliability of the DIO model.

### 2.4. Sample Preparation

After 8 weeks, each mouse was anesthetized with a ketamine-xylazine mixture (80–120 mg/kg) and intracardially perfused with sterilized cold saline and 4% paraformaldehyde. Brains were extracted, snap-frozen over liquid nitrogen, and stored at −80°C. A punch biopsy was performed, as described previously, to excise ARC tissue samples from the brain [10, 11], and the samples were deep-frozen immediately after sampling.

### 2.5. Protein Quantification

Each ARC sample was collected, as described previously [10, 11], and left and right samples were pooled and subjected to BCA/Bradford peptide assay for peptide extraction before quantification. Peptide sequences in the extracts were profiled in depth using a quadrupole-orbitrap mass spectrometer (Q Exactive Plus; Thermo Fisher Scientific, Waltham, MA, USA). Peptides were identified using the MaxQuant database (Max Planck Institute of Biochemistry, Munich, Germany) and assigned with a false discovery rate (FDR) < 1% [12]. The level of peptide expression was calculated using intensity-based absolute quantification (iBAQ) [13]. Peptides identified in at least one sample were included in the final list of valid proteins, which was imported to the Perseus platform (Max Planck Institute of Biochemistry) for statistical analyses and detection of differentially expressed proteins (DEPs).

### 2.6. Functional Pathway Analysis

The list of DEPs was uploaded via the STRING 11.0 tool to the STRING database (http://string-db.org) to generate a protein-protein interaction network (ELIXIR Core Data Resource) [14], and DEPs were searched against the Kyoto Encyclopedia of Genes and Genomes (KEGG) (http://www.kegg.jp/kegg/) and Gene Ontology (GO) (http://geneontology.org/) databases to evaluate functional enrichment. The overall characteristics of DEPs were examined by GO enrichment analysis [15] using the Biological Networks Gene Ontology (BiNGO) plug-in of Cytoscape [16]. Biochemical pathways involving two or more DEPs were determined [17].

### 2.7. Statistical Analysis

Bodyweight data are shown as means ± standard error of the mean and analyzed with two-way repeated measures analysis of variance implemented in SPSS (v.24.0; IBM Corp., Armonk, NY, USA).

For proteomic analysis for each protein, iBAQ intensities were transformed to log_2_ values, and missing values were imputed based on the Gaussian distribution. We then performed width adjustment (a process to equalize the quartiles of intensity values between the samples) for normalization. Student's *t*-tests were used for comparisons between the normalized iBAQ intensities of the HFD and LFD groups, and fold change values (defined as the ratio of the normalized mean iBAQ intensities of group HFD to group LFD) were then calculated. A DEP was defined as a protein with a *p* < 0.05 and either a fold change > 1.5 (upregulated protein) or <1.0/1.5 (downregulated protein). A volcano plot was generated for the DEPs based on *p* values and fold changes.

## 3. Results

### 3.1. Mouse Model of HFD-Induced Obesity

**Figure 1:**
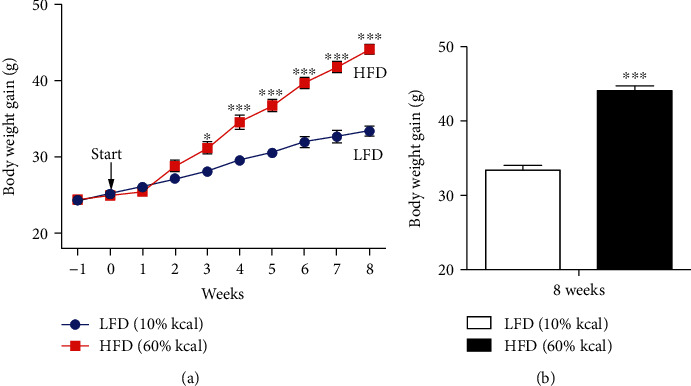
Mean change in body weight. (a) Mean body weights in the LFD (*n* = 5) and HFD (*n* = 5) groups were measured weekly from the adjustment period to the end of the experiment. (b) The mean body weight in the HFD group was significantly higher (*p* < 0.001) than that in the LFD group at the week of sacrifice (8 weeks after the adjustment period). ^∗^*p* < 0.05, ^∗∗∗^*p* < 0.001, two-way repeated measures analysis of variance. LFD: low-fat control diet; HFD: high-fat diet.

After unrestricted administration of an HFD for 8 weeks, a DIO model was successfully established. From 3 weeks (LFD vs. HFD: 28.06 ± 0.18 g vs. 31.16 ± 0.86 g, *F* = 12.501) to 8 weeks (LFD vs. HFD: 33.38 ± 0.64 g vs. 44.12 ± 0.62 g, *F* = 102.399) after the adjustment period, the mean weight in the HFD group was significantly higher than that in the LFD group (Figure 1(a)). After 15 weeks, the HFD group showed a significantly higher body weight than the LFD group (*p* < 0.0001) (Figure 1(b)).

### 3.2. Protein Quantification

**Figure 2:**
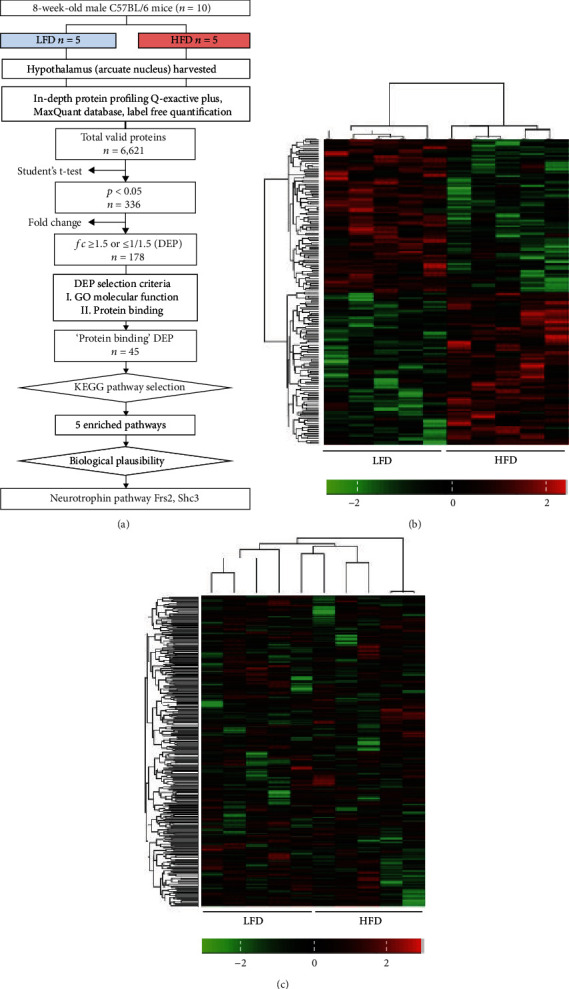
Pipeline and heatmap of DEPs. (a) A total of 178 proteins were differentially expressed between the two models. Heatmap of normalized iBAQ intensity values of (b) each DEP (total 178) and (c) each valid protein (total 6,621). Scale bars are shown on the bottom right corner of each image. Left columns represent the values from the LFD group, and right columns are from the HFD group. Hierarchical clustering analysis is based on Euclidean distances. DEP: differentially expressed protein; LFD: low-fat control diet; HFD: high-fat diet.

Left and right ARC tissue samples were pooled and used for peptide extraction and quantification. In total, 6,621 proteins were identified in the LFD and HFD groups. A volcano plot was generated to visualize the proteins and display the distribution and significant proteins (determined by −log_10_*p* values for the mean normalized iBAQ intensity). In total, 336 proteins were identified at a threshold *p* value of 0.05. After filtering with the fold change threshold, 178 proteins were identified as DEPs, including 89 upregulated DEPs (fold change ≥ 1.5) and 89 downregulated DEPs (fold change ≤ 0.66) (Figure 2(a), Table S1). Hierarchical clustering revealed a distinct proteomic signature in the hypothalamus under HFD and LFD. The normalized iBAQ intensities of the 178 DEPs and 6,621 valid proteins for each sample (five samples each for the LFD and HFD groups) were visualized using a heatmap (Figures 2(b) and 2(c)).

### 3.3. iBAQ Analysis of the Neurotrophin Signaling Pathway

**Figure 3:**
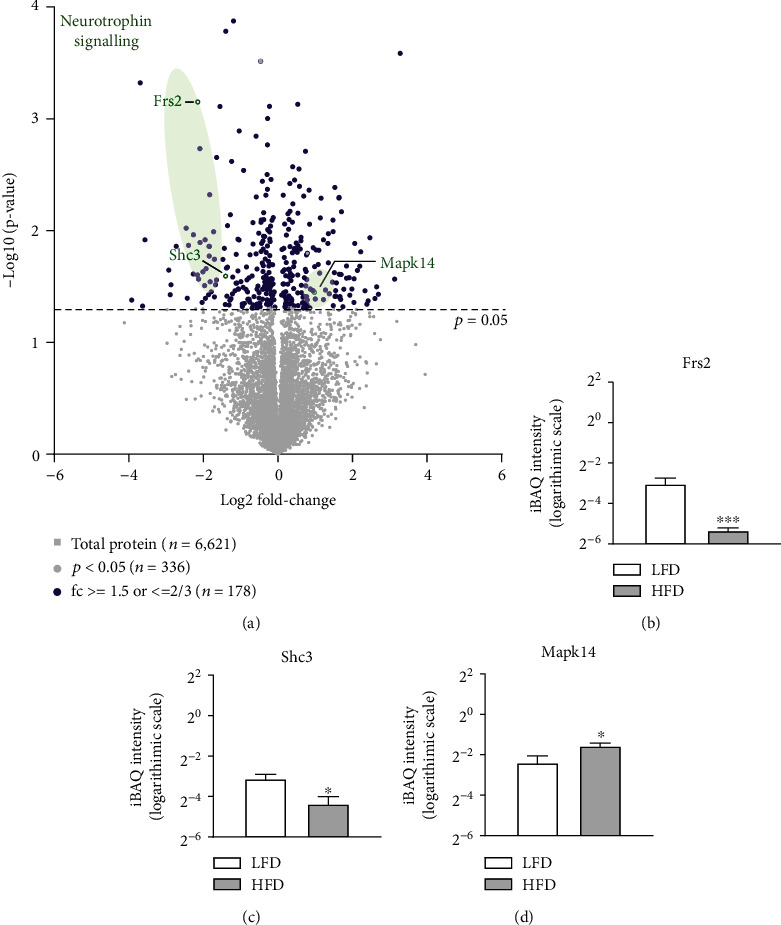
Volcano plot of DEPs. (a) The statistical significance (−log_10_*p* value between the mean iBAQ intensities of the LFD and HFD groups) is plotted against the difference in expression intensity (log_2_ fold change between the mean iBAQ intensities of the LFD and HFD groups) in a volcano plot. Green dots represent Frs2 and Shc3 in the neurotrophin signaling pathway. Data were filtered in accordance with the cut-off value of *p* < 0.05 and a fold change ≥ 1.5 or ≤1.0/1.5. Bar graphs indicate the mean and SEM of the iBAQ intensity (logarithmic scale) in each group of proteins. (b) Frs2 (*p* = 7.1 × 10^−4^, fold change = 0.26), (c) Shc3 (*p* = 0.03, fold change = 0.38), and (d) Mapk14 (*p* = 0.0753, fold change = 1.4146). LFD: low-fat control diet; HFD: high-fat diet; iBAQ: intensity-based absolute qualification; SEM: standard error of the mean; Shc3: SHC adaptor protein 3; Frs2: fibroblast growth factor receptor substrate 2; Mapk14: mitogen-activated protein kinase 14. ^∗^*p* < 0.05.

We evaluated a candidate pathway (the neurotrophin signaling pathway) based on previous results demonstrating its important roles in neurogenesis, axogenesis, and synaptic plasticity in the hypothalamus and feeding and energy imbalances due to obesity [18, 19]. Among the DEPs in our analysis, we detected two proteins (fibroblast growth factor (FGF) receptor substrate 2 (Frs2) and SHC adaptor protein 3 (Shc3)) involved in the neurotrophin signaling pathway (Figure 3(a)). The intensity-based quantification confirmed that Frs2 and Shc3 levels were lower in the HFD group than in the control group (Figures 3(b) and 3(c)).

### 3.4. KEGG Pathway Analysis

**Figure 4:**
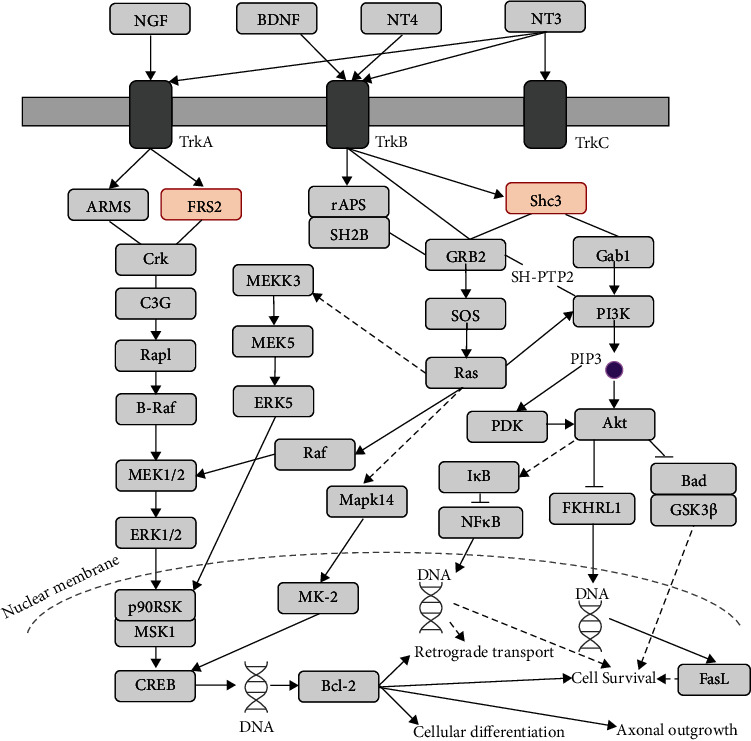
Neurotrophin signaling pathway and corresponding DEPs. “Neurotrophin signaling pathway” within the overall “full” KEGG pathway. DEP: differentially expressed protein; NGF: nerve growth factor; BDNF: brain-derived neurotrophic factor; NT3,4: neurotrophin 3,4; TrkA/B/C: neurotrophic receptor tyrosine kinase 1, 2, and 3; ARMS: ankyrin repeat-rich membrane-spanning protein; FRS2: fibroblast growth factor receptor substrate 2; CREB: CAMP-responsive element-binding protein; GRB2: growth factor receptor-bound protein 2; SOS: Son of Sevenless; Mapk14: mitogen-activated protein kinase 14; MK-2: mitogen-activated protein kinase-activated protein kinase 2; Gab1: GRB2-associated binding protein 1; PI3K: phosphoinositide-3-kinase; PIP3: phosphatidylinositol-3,4,5-trisphosphate; PDK: 3-phosphoinositide-dependent protein kinase-1; Bad: BCL2-associated agonist of cell death; GSK3*β*: glycogen synthase kinase 3 beta; FasL: Fas ligand.

**Figure 5:**
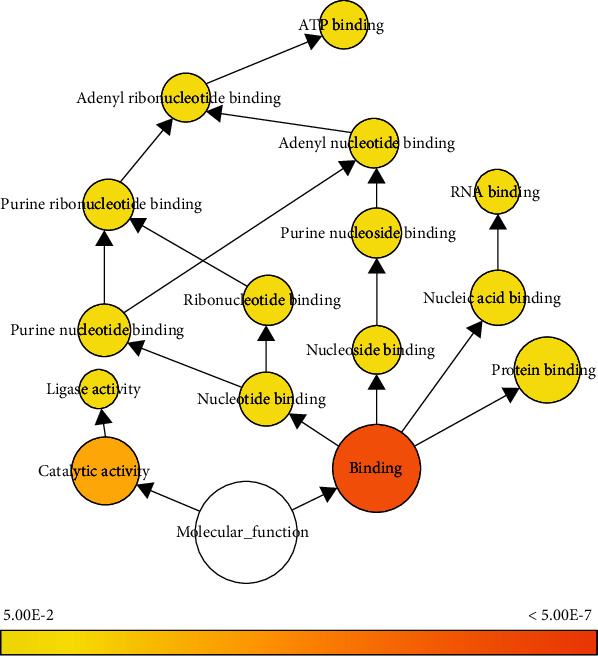
Molecular functions according to GO pathways associated with DEPs and visualized using the Biological Networks GO plug-in. Colored nodes correspond to GO terms that were significant according to a *p* = 0.05. Node sizes represent the number of DEPs related to the corresponding GO term. GO: Gene Ontology; DEP: differentially expressed gene.

The DEPs were further evaluated by KEGG pathway analysis, with a KEGG pathway diagram generated after deleting unrelated molecules and redundant data. Among 45 DEPs related to “protein binding,” five KEGG pathways that included ≥3 DEPs related to this term were further explored. The threshold was set to three DEPs, because the number of KEGG pathways was high (*n* = 16) when the threshold was set to two DEPs, which increased the risk of false-positive results. The five selected KEGG pathways and their corresponding genes were as follows: mitogen-activated protein kinase (MAPK) signaling pathway (Mapk14, Map3k12, and Rps6ka4), thermogenesis (Mapk14, Frs2, Prkab2, and Uqcrh), proteoglycans in cancer (Mapk14, Frs2, and Lum), *Salmonella* infection (Mapk14, Arpc5, and Wasf2), and neurotrophin signaling pathway (Mapk14, Frs2, and Shc3). The roles of the MAPK signaling pathway and the thermogenesis pathway are already clearly established; therefore, these pathways were excluded from further analyses. In addition, proteoglycans in cancer and *Salmonella* infection pathways were excluded, because they were highly unlikely to have therapeutic value in obesity. Accordingly, the neurotrophin signaling pathway (including Mapk14, Frs2, and Shc3) was selected as a key pathway for further analyses (Figure 4). The modified pathway diagram suggests that these proteins might suppress axonal outgrowth (Figure 5).

### 3.5. GO Functional Enrichment Analysis

According to GO enrichment analysis and the BiNGO network (Tables 1–3), DEPs were enriched for various GO terms and particularly in two categories: biological processes and cellular components (Figures S1 and S2). BiNGO enabled graphical visualization of several DEPs and *p* values for each GO term (Figure 4). The results showed that enriched terms in the molecular function category included “binding” (*p* = 3.9 × 10^−7^, 90 DEPs) and catalytic activity (*p* = 6.9 × 10^−4^, 47 DEPs) (Figure 4). Compared with catalytic activity, “binding” included more DEPs and was more highly significant. Among subgroups of the “binding” term, “protein binding” (*p* = 0.02, 45 DEPs) was the most highly significant and included the most DEPs. The DEPs related to “protein binding” were further evaluated for involvement in KEGG pathways, revealing one pathway related to the hypothalamic ARC (i.e., the neurotrophin signaling pathway) (Figure 4).

## 4. Discussion

This study identified DEPs in the ARC between mice with HFD-induced obesity and mice fed an LFD. Glutamate transporters (e.g., GLT and GLAST) are among the exceptionally abundant and important proteins in the central nervous system (~>2.5% of the brain protein mass exists in the form of glutamate transporters) and critical for the function of glutamatergic signaling (the balance between glutamate excitation and GABA inhibition is particularly important and relevant for body mass control mechanisms in the hypothalamus). Although proteomic changes in the hypothalamus of an obesity model have been studied [9], our comparison of the ARC in LFD and HFD groups was novel. Moreover, in-depth profiling of the global proteome targeting the ARC using the punch biopsy method revealed various proteins that have not previously been reported in studies of obesity or energy metabolism.

Recent studies suggest that tumor necrosis factor *α* and interleukin 1*β*, especially in AgRP-producing neurons, might contribute to inflammation-induced anorexia during acute inflammatory conditions [20]. Additionally, single-cell RNA sequencing of the hypothalamic ARC after HFD-induced obesity demonstrated selective changes in AgRP neurons via neuron-astrocyte interactions, which contributed to the exaggerated sympathoexcitation observed in obese rats [21, 22]. These effects might be mediated by the regulatory effects of leptin on astrocytic glutamate transporters within the ARC of the hypothalamus [23].

GO enrichment analysis identified binding and catalytic activity as key molecular functions. Notably, the DEPs related to protein binding were involved in the neurotrophin signaling pathway. These results were consistent with those of previous reports indicating that the neurotrophin signaling pathway contributes to neurogenesis, axogenesis, and synaptic plasticity in the hypothalamus and dysfunctions in feeding and energy balance due to obesity [18, 19]. “Binding” was a highly significant term and included a number of DEPs, and the subgroup “protein binding” was further identified as a significant molecular function. DEPs associated with “protein binding,” particularly Frs2 and Shc3, were involved in the neurotrophin signaling pathway. This pathway is generally involved in retrograde transport, cell survival, cellular differentiation, and axonal outgrowth of neurons and triggered by the binding of extracellular signaling molecules, such as nerve growth factor, brain-derived neurotrophic factor (BDNF), and neurotrophins 4 and 3. Furthermore, a previous study reported that the distribution of tropomyosin receptor kinase B- (TrkB-) expressing neurons is altered in response to an HFD in the mediobasal hypothalamus [24].

These results showed that Frs2 and Shc3 levels and the related neurotrophin pathway were altered in the ARC of a mouse model of HFD-induced obesity. Neurotrophins, which mediate receptors and signaling molecules, play multiple roles in survival and development [25]. FRS2 functions by transmitting FGF and/or neurotrophin signaling during dorsal forebrain development, with a critical role in the formation of the hippocampal dentate gyrus [26]. Additionally, *ARC^TrkB^* neurons regulate food intake by projecting to BDNF-expressing neurons in the PVN [27].

A previous study reported that BDNF deficiency signaled a reduced number of axonal projections of *ARC^TrkB^* to PVN, subsequently inducing hyperphagia [28]. SHC molecules and neurotrophins bind to Trks and the neurotrophin receptor [25, 29]. In the present study, the DEPs included Frs2 and Shc3, which represent novel candidate pharmaceutical targets for obesity based on their close links to pathways involved in obesity. Furthermore, these two proteins identified by in-depth profiling of the ARC using the punch biopsy method have not previously been reported in studies of obesity or energy metabolism. However, the central effects of Frs2 or Shc3 remain unknown. To fully understand their roles in the regulation of energy expenditure and food intake, further studies are needed, such as an intracerebroventricular injection study along with analyses of hourly and daily food intake and metabolic parameters.

## 5. Conclusions

We identified 178 DEPs in the ARC of a reliable mouse model of HFD-induced obesity. These DEPs play key roles in protein binding and the neurotrophin signaling pathway and include Frs2 and Shc3 as novel candidates involved in appetite and energy metabolism.

## Figures and Tables

**Table 1:** GO pathway analysis—biological process.

Term (GO ID)	Reference	Observed	Expected	F.E.	*p* value	FDR
Negative regulation of protein kinase activity by regulation of protein phosphorylation (0044387)	8	3	0.06	47.78	7.32*E* − 05	3.99*E* − 02
mRNA catabolic process (0006402)	119	7	0.93	7.49	5.97*E* − 05	3.37*E* − 02
RNA metabolic process (0016070)	1234	25	9.69	2.58	1.56*E* − 05	1.03*E* − 02
Nucleic acid metabolic process (0090304)	1770	34	13.89	2.45	1.58*E* − 06	1.39*E* − 03
Nucleobase-containing compound metabolic process (0006139)	2194	41	17.22	2.38	1.55*E* − 07	3.05*E* − 04
Organic cyclic compound metabolic process (1901360)	2618	46	20.55	2.24	1.92*E* − 07	3.37*E* − 04
Organic substance metabolic process (0071704)	6707	89	52.64	1.69	1.16*E* − 08	4.56*E* − 05
Metabolic process (0008152)	7224	98	56.7	1.73	1.94*E* − 10	1.54*E* − 06
Cellular nitrogen compound metabolic process (0034641)	2791	46	21.91	2.1	9.02*E* − 07	8.90*E* − 04
Nitrogen compound metabolic process (0006807)	5727	79	44.95	1.76	2.98*E* − 08	9.41*E* − 05
Cellular metabolic process (0044237)	6339	93	49.75	1.87	1.01*E* − 11	1.59*E* − 07
Cellular process (0009987)	14067	143	110.41	1.3	1.40*E* − 07	3.15*E* − 04
Heterocycle metabolic process (0046483)	2331	42	18.3	2.3	3.27*E* − 07	5.17*E* − 04
Cellular aromatic compound metabolic process (0006725)	2405	41	18.88	2.17	1.96*E* − 06	1.55*E* − 03
Primary metabolic process (0044238)	6275	88	49.25	1.79	1.00*E* − 09	5.28*E* − 06
Macromolecule metabolic process (0043170)	5065	70	39.75	1.76	4.34*E* − 07	5.71*E* − 04
Nucleobase-containing compound catabolic process (0034655)	234	13	1.84	7.08	7.58*E* − 08	2.00*E* − 04
Aromatic compound catabolic process (0019439)	292	13	2.29	5.67	8.47*E* − 07	8.92*E* − 04
Cellular nitrogen compound catabolic process (0044270)	272	13	2.13	6.09	3.94*E* − 07	5.66*E* − 04
Organic cyclic compound catabolic process (1901361)	323	13	2.54	5.13	2.48*E* − 06	1.87*E* − 03
Heterocycle catabolic process (0046700)	278	13	2.18	5.96	4.99*E* − 07	6.06*E* − 04
Cellular macromolecule metabolic process (0044260)	3967	58	31.14	1.86	1.31*E* − 06	1.22*E* − 03
rRNA processing (0006364)	199	9	1.56	5.76	3.84*E* − 05	2.25*E* − 02
Cellular component organization or biogenesis (0071840)	5284	67	41.47	1.62	2.29*E* − 05	1.45*E* − 02
rRNA metabolic process (0016072)	208	10	1.63	6.13	8.53*E* − 06	6.13*E* − 03
ncRNA metabolic process (0034660)	419	15	3.29	4.56	1.67*E* − 06	1.39*E* − 03
ncRNA processing (0034470)	343	12	2.69	4.46	2.35*E* − 05	1.43*E* − 02
Cellular biosynthetic process (0044249)	1984	34	15.57	2.18	1.46*E* − 05	1.00*E* − 02
Biosynthetic process (0009058)	2157	34	16.93	2.01	9.15*E* − 05	4.67*E* − 02
G protein-coupled receptor signaling pathway (0007186)	1849	0	14.51	<0.01	5.21*E* − 07	5.88*E* − 04
Sensory perception of chemical stimulus (0007606)	1225	0	9.61	<0.01	8.20*E* − 05	4.32*E* − 02

GO: Gene Ontology; FDR: false discovery rate; F.E.: fold enrichment.

**Table 2:** GO pathway analysis—cellular component.

Term (GO ID)	Reference	Observed	Expected	F.E.	*p* value	FDR
Small-subunit processome (0032040)	37	5	0.29	17.22	1.82*E* − 05	3.01*E* − 03
Preribosome (0030684)	81	6	0.64	9.44	6.13*E* − 05	7.16*E* − 03
Protein-containing complex (0032991)	5312	64	41.69	1.54	1.69*E* − 04	1.59*E* − 02
Nucleolus (0005730)	808	19	6.34	3	2.58*E* − 05	3.66*E* − 03
Nuclear lumen (0031981)	3906	60	30.66	1.96	1.22*E* − 07	3.02*E* − 05
Intracellular organelle lumen (0070013)	4314	64	33.86	1.89	1.31*E* − 07	2.88*E* − 05
Organelle lumen (0043233)	4315	64	33.87	1.89	1.31*E* − 07	2.61*E* − 05
Membrane-enclosed lumen (0031974)	4315	64	33.87	1.89	1.31*E* − 07	2.37*E* − 05
Cellular anatomical entity (0110165)	18664	170	146.49	1.16	2.31*E* − 08	6.56*E* − 06
Organelle (0043226)	12317	140	96.68	1.45	1.14*E* − 11	2.27*E* − 08
Intracellular organelle (0043229)	11971	135	93.96	1.44	2.06*E* − 10	2.05*E* − 07
Intracellular (0005622)	13708	146	107.59	1.36	3.61*E* − 10	2.39*E* − 07
Nucleus (0005634)	6832	79	53.62	1.47	6.93*E* − 05	7.24*E* − 03
Intracellular membrane-bounded organelle (0043231)	10296	119	80.81	1.47	7.41*E* − 09	2.45*E* − 06
Membrane-bounded organelle (0043227)	11045	127	86.69	1.46	1.05*E* − 09	5.21*E* − 07
Intracellular nonmembrane-bounded organelle (0043232)	4175	55	32.77	1.68	5.81*E* − 05	7.20*E* − 03
Nonmembrane-bounded organelle (0043228)	4194	55	32.92	1.67	6.20*E* − 05	6.84*E* − 03
Mitochondrion (GO:0005739)	1798	30	14.11	2.13	1.22*E* − 04	1.22*E* − 02
Cytoplasm (0005737)	10948	126	85.93	1.47	1.10*E* − 09	4.37*E* − 07
Cytosol (0005829)	3534	50	27.74	1.8	2.57*E* − 05	3.92*E* − 03
Nucleoplasm (0005654)	3331	47	26.14	1.8	4.51*E* − 05	5.97*E* − 03

GO: Gene Ontology; FDR: false discovery rate; F.E.: fold enrichment.

**Table 3:** GO pathway analysis—molecular function.

Term (GO ID)	Reference	Observed	Expected	F.E.	*p* value	FDR
RNA binding (0003723)	1091	23	8.56	2.69	1.92*E* − 05	2.96*E* − 02
Binding (0005488)	13336	133	104.67	1.27	8.63*E* − 06	1.99*E* − 02
Transmembrane signaling receptor activity (0004888)	2135	2	16.76	0.12	7.22*E* − 06	3.33*E* − 02

GO: Gene Ontology; FDR: false discovery rate; F.E.: fold enrichment.

## Data Availability

The data used to support the findings of this study are included within the article.

## Supplementary Materials

Supplementary 1 Figure S1: GO pathway—biological process. Figure S2: GO pathway—cellular component.

Supplementary 2 Table S1: DEPs and iBAQ intensity values in the HFD group relative to the LFD group.
