# Supplementary material for: Proteome Analysis of the Hypothalamic Arcuate Nucleus in Chronic High-Fat Diet-Induced Obesity
Source: Biomed Res Int. 2021 Nov 18;2021:3501770. doi: 10.1155/2021/3501770 (PMC8617565; doi:10.1155/2021/3501770)
Supplement: Supplementary 1 — Figure S1: GO pathway—biological process. Figure S2: GO pathway—cellular component. [file 3501770.f1.docx]

**Supplementary Materials**


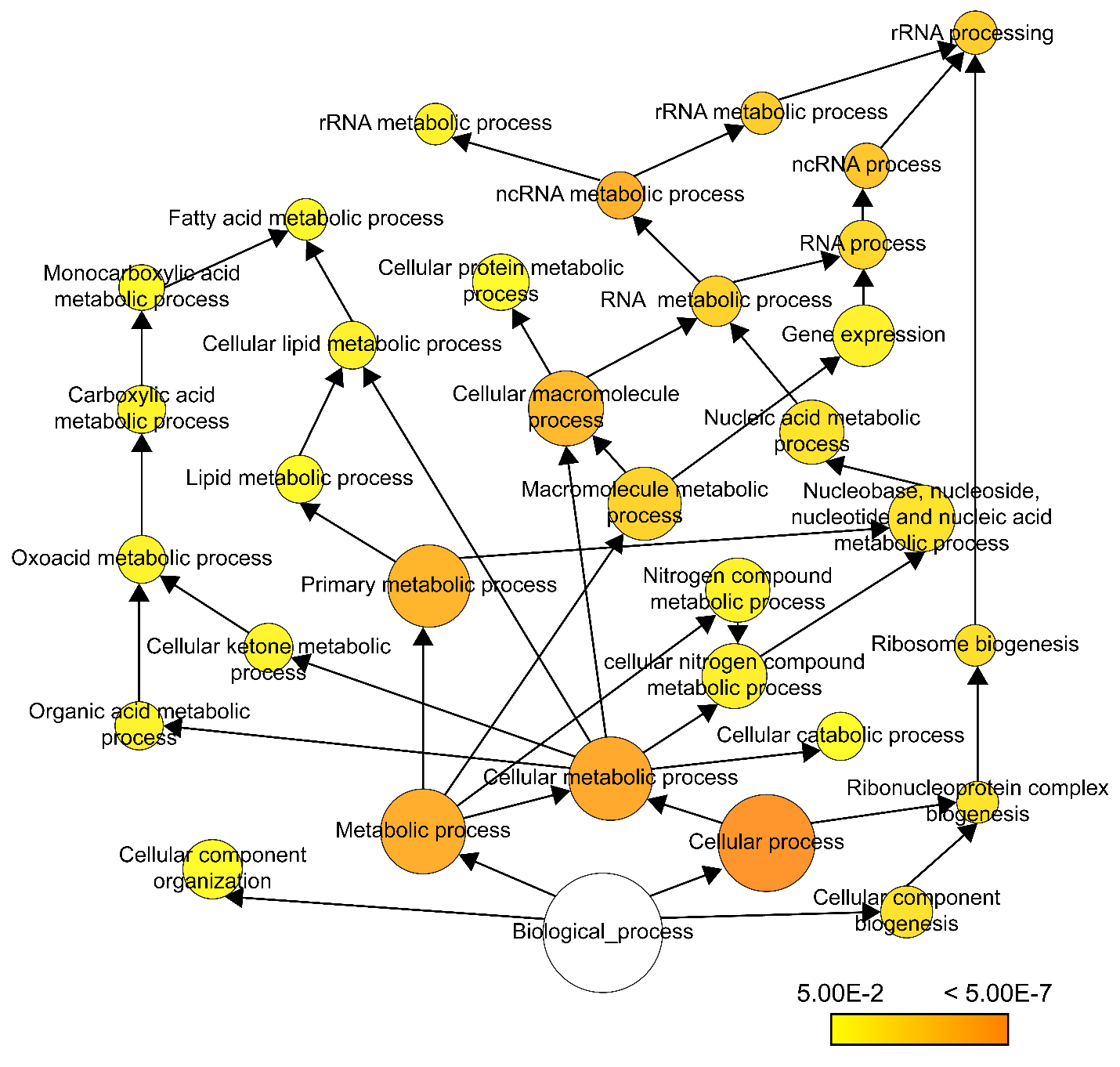


**Figure S1.** GO pathway: biological process. Enriched Gene Ontology (GO) pathways classified as biological processes are shown as a diagram generated using the Biological Networks Gene Ontology plug-in. Colored nodes correspond to GO terms that were significant according to the p-value (p = 0.05). The size of the node represents the number of DEPs associated with the corresponding GO term.


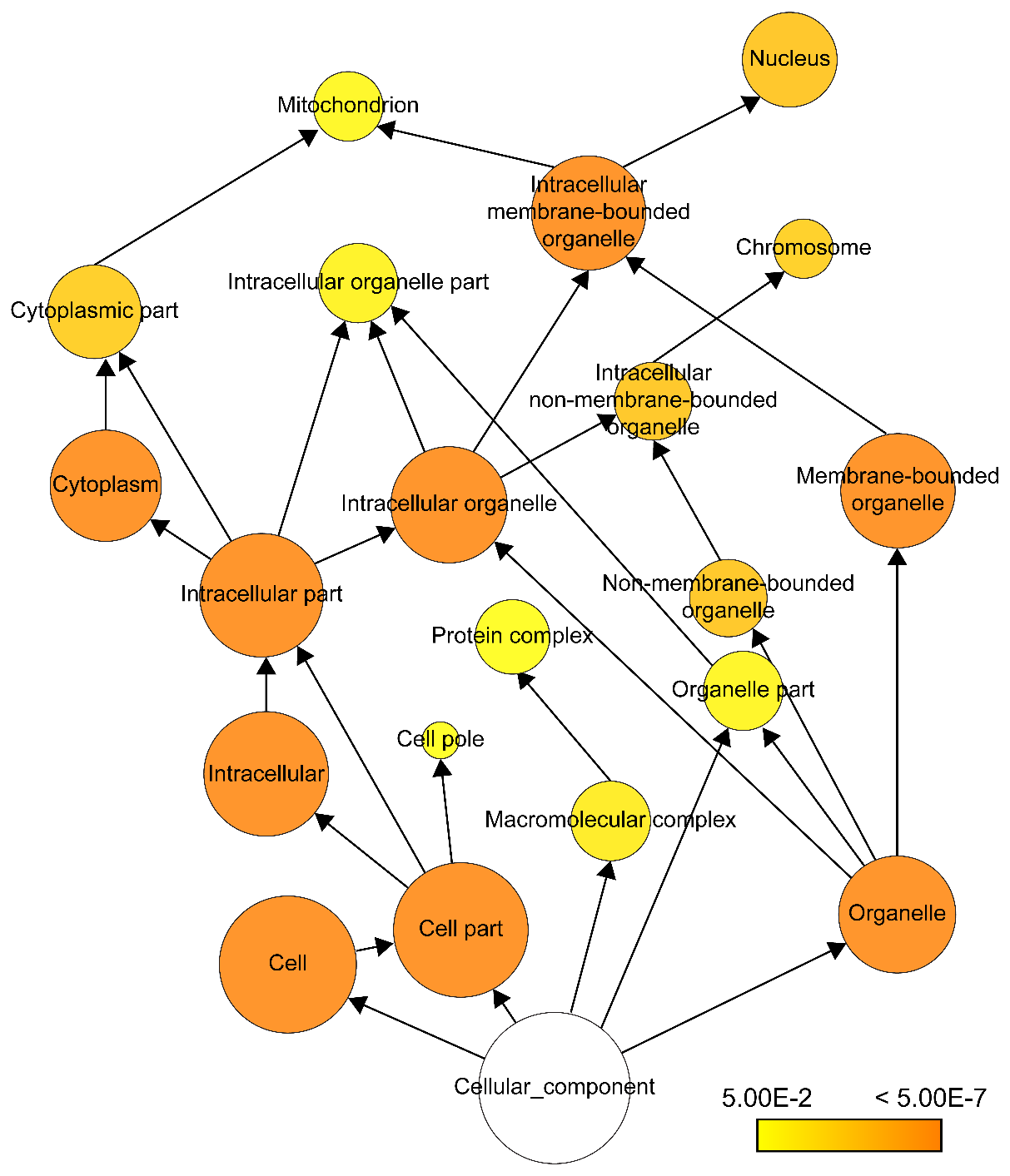


**Figure S2.** Gene Ontology (GO) pathway: cellular component. Enriched GO pathways classified as cellular components are shown as a diagram generated using the Biological Networks Gene Ontology plug-in. Colored nodes correspond to GO terms that were significant according to the p-value. The size of the node represents the number of DEPs associated with the corresponding GO term.
