# Supplementary material for: Proteome Analysis of the Hypothalamic Arcuate Nucleus in Chronic High-Fat Diet-Induced Obesity
Source: Biomed Res Int. 2021 Nov 18;2021:3501770. doi: 10.1155/2021/3501770 (PMC8617565; doi:10.1155/2021/3501770)
Supplement: Supplementary 2 — Table S1: DEPs and iBAQ intensity values in the HFD group relative to the LFD group. [file 3501770.f2.docx]

Table S1. DEPs and iBAQ intensity values in the HFD group relative to the LFD group.

| Majority protein IDs | Protein names | T: Gene names | iBAQ HFD1 | iBAQ HFD2 | iBAQ LFD1 | iBAQ LFD2 | N: Peptides | N: Razor + unique peptides | N: Unique peptides | N: Sequence coverage [%] |
| --- | --- | --- | --- | --- | --- | --- | --- | --- | --- | --- |
| Q7TT23;Q8BIT3;Q3TYT7;  Q3V0A7 | Uncharacterized protein C20orf194 homolog | 4930402H24Rik | −6.45485 | −5.44035 | −4.92543 | −5.73687 | 5 | 5 | 5 | 6.1 |
| A2AJ26;P41234;Q6ZPZ4 | ATP-binding cassette sub­family A member 2 | Abca2 | −2.98876 | −3.12958 | −3.58986 | −4.29994 | 20 | 20 | 19 | 11.8 |
| Q922Z5;Q9DBL9-  2;Q9DBL9 | 1-acylglycerol-3-phosphate O- acyltransferase ABHD5 | Abhd5 | −3.24559 | −2.86178 | −3.19113 | −5.45905 | 1 | 1 | 1 | 11.3 |
| Q91V12-4 | Cytosolic acyl coenzyme A thioester hydrolase | Acot7 | −6.59433 | −5.63279 | −3.23024 | −3.24282 | 19 | 1 | 1 | 61.2 |

| Q14DH7;Q14DH7-2 | Acyl-CoA synthetase short-chain family member 3, mitochondrial | Acss3 | −4.67691 | −3.7834 | −4.56841 | −5.22437 | 6 | 6 | 5 | 10.4 |
| --- | --- | --- | --- | --- | --- | --- | --- | --- | --- | --- |
| Q91ZS8-5;Q91ZS8-  4;Q91ZS8-3;Q91ZS8-  2;Q91ZS8;Q91ZS8-6 | Double-stranded RNA-specific editase 1 | Adarb1 | −5.10971 | −4.58754 | −4.50032 | −3.73146 | 7 | 7 | 7 | 18.3 |
| Q3U1U7-2;Q3U1U7 | Aryl hydrocarbon receptor | Ahrr | 1.85911 | 0.771552 | 1.24295 | 3.82732 | 1 | 1 | 1 | 3.5 |
|  | repressor |  |  |  |  |  |  |  |  |  |
| Q8K1C0-5;Q8K1C0- |  |  |  |  |  |  |  |  |  |  |
| 4;Q8K1C0-3;Q8K1C0- | Protein angel homolog 2 | Angel2 | −2.09391 | −1.56357 | −2.58608 | −3.31804 | 1 | 1 | 1 | 8.5 |
| G5E832;D3YTR0;Q9Z1K7 | Adenomatous polyposis coli protein 2 | Apc2 | −6.33059 | −6.77035 | −5.72613 | −4.58439 | 12 | 11 | 11 | 7.4 |
| Q3UC32 | Actin-related protein 2/3 | Arpc5 | −1.20436 | −1.92973 | −4.07197 | −3.87046 | 5 | 1 | 1 | 38.4 |
|  | complex subunit 5 |  |  |  |  |  |  |  |  |  |
| Q499E0 | BMP/retinoic acid-inducible | Brinp3 | −5.90346 | −2.81933 | −2.08938 | −2.04048 | 6 | 5 | 3 | 7.8 |
|  | neural-specific protein 3 |  |  |  |  |  |  |  |  |  |
| E9Q7P2;E0CXK2 |  | Cacna1i | −5.16306 | −6.389 | −4.04982 | −4.24719 | 7 | 7 | 6 | 3.8 |
| Q8BYM8;G3X975 | Probable cysteine-tRNA | Cars2 | 0 | 0.0775786 | −1.32234 | −1.60216 | 11 | 11 | 11 | 25 |
|  | ligase, mitochondrial |  |  |  |  |  |  |  |  |  |
| P83917;Q7TPM0;Q9CYJ8 | Chromobox protein homolog 1 | Cbx1 | −1.5047 | −1.07544 | −4.10307 | −4.53493 | 3 | 2 | 2 | 18.4 |
| F6UK66;Q3TNK7;A6H6M8 | Coiled-coil domain-containing |  |  |  |  |  |  |  |  |  |
| ;Q810U5-3;Q810U5- |  | Ccdc50 | −4.29568 | −6.02131 | −2.12004 | −2.92541 | 2 | 2 | 2 | 6.2 |
| 2;Q810U5 | protein 50 |  |  |  |  |  |  |  |  |  |
| Q61735-2 | Leukocyte surface antigen CD47 | Cd47 | 3.09659 | 3.2438 | 1.96999 | 1.46705 | 6 | 6 | 2 | 21.9 |
| Q8VI68;P70408;H3BKV8 | Cadherin-10 | Cdh10 | −4.28612 | −3.77909 | −2.91329 | −2.74822 | 3 | 3 | 3 | 4.4 |
| Q3U506;Q3TLJ5;Q99LM2; | CDK5 regulatory subunit- | Cdk5rap3 | 0.0821046 | −0.449861 | −2.0775 | −0.98623 | 14 | 14 | 14 | 23.9 |
| Q3TSS9 | associated protein 3 |  |  |  |  |  |  |  |  |  |
| Q924Z4;D3Z4M2;D3YTM0  ;D3Z0Z2 | Ceramide synthase 2 | Cers2 | −1.02575 | −0.740997 | −2.25429 | −4.98263 | 3 | 3 | 3 | 10 |
|  | Chloride channel |  |  |  |  |  |  |  |  |  |
| Q3TBI1;A9C437;Q9R0A1 | protein;Chloride channel | Clcn2 | −4.20101 | −3.43881 | −3.4112 | −5.19933 | 7 | 7 | 6 | 10.3 |
|  | protein 2 |  |  |  |  |  |  |  |  |  |
| Q5SW19;Q5SW19- | Clustered mitochondria protein | Cluh | −0.619007 | −0.877093 | −0.0653113 | 0.0137 | 27 | 27 | 3 | 24.4 |
| 3;Q5SW19-2 | homolog |  |  |  |  |  |  |  |  |  |
| Q543X5;Q3V231;Q60809; | CCR4-NOT transcription | Cnot7 | −0.559772 | −1.70978 | −3.64713 | −6.4972 | 3 | 3 | 3 | 14.4 |
| Q3TLK9 | complex subunit 7 |  |  |  |  |  |  |  |  |  |
| Q8CBC4;Q8CBC4-3 | Consortin | Cnst | −5.33589 | −1.84148 | −2.75675 | −2.46807 | 4 | 4 | 4 | 6.3 |
| Q3UM29;Q3TR20 | Conserved oligomeric Golgi complex subunit 7 | Cog7 | −2.61279 | −1.58304 | −4.1922 | −2.75716 | 10 | 10 | 10 | 16.2 |
| Q3TIJ1 |  | Cops4 | −4.68338 | −4.88599 | −5.10166 | −5.03306 | 23 | 1 | 1 | 75.6 |
| Q5SUQ9;Q5SUQ9-3 | CST complex subunit CTC1 | Ctc1 | −3.95641 | −5.8249 | −2.84026 | −2.46046 | 11 | 11 | 1 | 13.4 |
| D5MCW4;Q9CQ89;Q8VC 83;REV__Q8CEV3;REV__ Q3TR22;REV__Q8CEC8; REV__Q8BNP6;REV__Q7 TPC7;REV__O08796;G3X | Protein CutA | Cuta;Skida1 | 1.70172 | 1.6477 | 3.15627 | 3.47057 | 2 | 2 | 1 | 13 |
|  |  |  |  |  |  |  |  |  |  |  |
| 9M2;Q9CQ89-3 |  |  |  |  |  |  |  |  |  |  |
| D3Z7F1 |  | Cyp2s1 | −4.22889 | −2.93228 | −1.76493 | −2.39175 | 7 | 7 | 1 | 20.5 |
| F6VRL0;F2Z465;B2RRQ8; |  |  |  |  |  |  |  |  |  |  |
| P97318-4;P97318-  5;P97318-6;P97318-  3;P97318-8;P97318- | Disabled homolog 1 | Dab1 | −3.01789 | −5.51368 | −2.8385 | −1.78622 | 1 | 1 | 1 | 8.4 |
| 2;P97318 |  |  |  |  |  |  |  |  |  |  |
| Q3TYA0;D3Z4U8;Q3UD98 | DDB1- and CUL4-associated | Dcaf11 | −3.37567 | −1.94034 | −1.15178 | −1.50235 | 5 | 5 | 5 | 12.9 |
| ;Q91VU6-2;Q91VU6 | factor 11 |  |  |  |  |  |  |  |  |  |
| Q8K0V2;D3YWG7 | DCN1-like protein 3;DCN1-like protein | Dcun1d3 | −1.41208 | −0.73413 | −2.91565 | −2.85755 | 5 | 5 | 5 | 17.8 |
| Q9CXR1 | Dehydrogenase/reductase SDR | Dhrs7 | 0.318033 | 0.769068 | 0.0111242 | 0.270799 | 9 | 9 | 9 | 35.5 |
|  | family member 7 |  |  |  |  |  |  |  |  |  |
| D6RI31;Q8BFQ6 | Disrupted in renal carcinoma protein 2 homolog | Dirc2 | −6.48812 | −6.76399 | 0.21783 | 0.85547 | 1 | 1 | 1 | 13 |
| Q80TN4 | DnaJ homolog subfamily C member 16 | Dnajc16 | −1.2273 | −1.92571 | 0.681585 | −1.36294 | 13 | 13 | 13 | 23.7 |
| Q8JZM4 | Delta and Notch-like epidermal growth factor-related receptor | Dner | −6.92528 | −2.43439 | −1.68129 | −0.989163 | 4 | 4 | 4 | 7.1 |
| Q80VJ3 | 2-deoxynucleoside 5- phosphate N-hydrolase 1 | Dnph1 | −5.99192 | −6.70502 | −4.28463 | −2.2249 | 3 | 3 | 3 | 27.2 |
| Q5NBZ3;P32233;Q8CHR8 | Developmentally-regulated | Drg1 | −1.91091 | −1.9085 | −0.87949 | −1.38737 | 11 | 10 | 10 | 37.9 |
| ;Q8BNS0 | GTP-binding protein 1 |  |  |  |  |  |  |  |  |  |
| Q9D700 | Dual specificity protein | Dusp26 | −5.81787 | −4.81482 | −2.35325 | −3.91847 | 2 | 2 | 2 | 11.4 |
|  | phosphatase 26 |  |  |  |  |  |  |  |  |  |
| Q3UN47;O08600 | Endonuclease G, mitochondrial | Endog | −1.15211 | −0.437975 | −0.0883909 | 0.202813 | 9 | 9 | 9 | 45.6 |
| Q5DTL0;Q9EQG7;Q5FWJ  8 | Ectonucleotide  pyrophosphatase/phosphodiest | Enpp5 | −3.35309 | −3.02142 | −2.71902 | −1.65777 | 9 | 9 | 9 | 28.2 |
|  | erase family member 5 |  |  |  |  |  |  |  |  |  |
|  | EGF domain-specific O-linked |  |  |  |  |  |  |  |  |  |
| Q8BYW9 | N-acetylglucosamine | Eogt | −3.33962 | −3.32929 | −3.82491 | −4.09723 | 7 | 7 | 7 | 14.2 |
|  | transferase |  |  |  |  |  |  |  |  |  |
| A2ALK6;Q9JMC8;Q3TMR  3 | Band 4.1-like protein 4B | Epb4.1l4b;Epb41l4b | −1.13959 | −1.22819 | −2.6594 | −1.65524 | 12 | 12 | 10 | 18.1 |
| Q8BTW3 | Exosome complex component MTR3 | Exosc6 | −1.00937 | −0.782271 | −1.77836 | −1.8389 | 4 | 4 | 4 | 23.4 |

| B7ZMR0;Q76LS9-  2;Q76LS9 | Protein FAM63A | Fam63a | −1.44312 | −1.12313 | −0.667263 | −0.251995 | 5 | 5 | 5 | 18.2 |
| --- | --- | --- | --- | --- | --- | --- | --- | --- | --- | --- |
| Q6PDI6-3;Q6PDI6 | Protein FAM63B | Fam63b | −0.125214 | 0.223038 | −1.48128 | −0.414883 | 6 | 6 | 6 | 16.5 |
| Q8BK03;Q8BK03-2 | Protein FAM73B | Fam73b | −1.69952 | −2.84606 | −1.55983 | −1.9373 | 9 | 9 | 9 | 18.4 |
| Q9D187 | Mitotic spindle-associated MMXD complex subunit MIP18 | Fam96b | 1.12756 | −6.21802 | 1.45766 | 0.952637 | 3 | 3 | 3 | 28.2 |
| E9QK16;Q8BNA6;Q8BNA  6-2 | Protocadherin Fat 3 | Fat3 | −6.27364 | −5.5624 | −6.35879 | −7.55215 | 4 | 4 | 4 | 1 |
| Q3USJ8-  3;Q3USJ8;Q3USJ8-2 | F-BAR and double SH3 domains protein 2 | Fchsd2 | −3.53575 | −6.10973 | −3.07717 | −1.72004 | 7 | 7 | 7 | 12.8 |
| Q3TS40;P53798 | Squalene synthase | Fdft1 | −3.46971 | −2.81677 | −2.76773 | −5.43958 | 1 | 1 | 1 | 2.9 |
| A2AR56;Q6P5H6;B0R0D5  ;B0R0D4;Q6P5H6-  2;Q8BXT6;F6XM46;Q6P5  H6-4;Q6P5H6- | FERM domain-containing protein 5 | Frmd5 | −3.14557 | −4.34071 | −2.75098 | −2.05509 | 9 | 9 | 9 | 19.6 |
| 3;Q8BX61;Q8BW31 |  |  |  |  |  |  |  |  |  |  |
| Q8C180 | Fibroblast growth factor receptor substrate 2 | Frs2 | −5.66726 | −5.43548 | −3.05839 | −2.25541 | 6 | 4 | 4 | 15.6 |
| Q3TLS3 | GDP-D-glucose phosphorylase  1 | Gdpgp1 | −0.359084 | −0.764789 | 0.0661494 | 0.317993 | 10 | 10 | 10 | 32.4 |
| Q80Y14 | Glutaredoxin-related protein 5, mitochondrial | Glrx5 | 3.23625 | 3.19602 | 1.06607 | 1.02876 | 3 | 3 | 3 | 25.7 |
| 2;Q80TI0- | GRAM domain-containing protein 1B | Gramd1b | −2.85204 | −3.05268 | −2.47131 | −1.92152 | 7 | 7 | 6 | 11.3 |
| O09131 | Glutathione S-transferase omega-1 | Gsto1 | 5.28462 | 3.98028 | 3.41925 | 3.21194 | 13 | 13 | 13 | 59.2 |
| Q8C3X4;Q8C3X4−  2;G3UWY0;Q8C8D6;F6Z  M03 | Translation factor Guf1, mitochondrial | Guf1 | −3.62172 | −3.09288 | −3.96326 | −5.12859 | 8 | 8 | 8 | 16.9 |
| Q3UMU9-4;Q3UMU9-  2;Q3UMU9;Q3UMU9-3 | Hepatoma-derived growth factor-related protein 2 | Hdgfrp2 | −0.845588 | −0.473988 | −2.31672 | −2.27484 | 7 | 5 | 5 | 13.5 |
| Q1WWK3;P43276 | Histone H1.5 | Hist1h1b | 3.99326 | 3.88545 | 4.10302 | 3.25317 | 4 | 4 | 4 | 17.6 |
| Q3TC93 | HCLS1-binding protein 3 | Hs1bp3 | −1.1876 | −2.15613 | −0.475672 | −0.359586 | 4 | 4 | 4 | 17 |
| Q8R035;Q8R035-  2;A2A6T4 | Peptidyl-tRNA hydrolase ICT1, mitochondrial | Ict1 | −0.429551 | −0.423852 | −5.05519 | −2.50132 | 6 | 6 | 6 | 32.5 |
| Q6P9J5 | KN motif and ankyrin repeat domain-containing protein 4 | Kank4 | −6.91744 | −6.64914 | −3.10735 | −4.38799 | 6 | 6 | 6 | 9.3 |
| G5E897;Q8C6F3 |  | Kdelc2 | −2.28353 | −2.70139 | −1.21065 | −0.305805 | 7 | 7 | 7 | 17.7 |
| B2RUK8;Q3USL1 | Kelch domain-containing protein 9 | Klhdc9 | −2.28816 | −2.57409 | −2.52068 | −3.29215 | 2 | 2 | 2 | 9.7 |
| Q8C7P1;Q6ZPT1 | Kelch-like protein 9 | Klhl9 | −5.15229 | −5.40777 | −3.84792 | −5.13878 | 3 | 3 | 3 | 5.8 |
| P11679 | Keratin, type II cytoskeletal 8 | Krt8 | −2.32536 | −3.38929 | −5.82073 | −4.25728 | 4 | 3 | 1 | 9.4 |
| Q07797 | Galectin-3-binding protein | Lgals3bp | −2.90559 | −2.43736 | −1.5894 | −3.45261 | 8 | 8 | 8 | 20.3 |
| Q6PDR1;Q3TEL5;Q9Z0M  5 | Lipase;Lysosomal acid lipase/cholesteryl ester | Lipa | −4.47195 | −4.38761 | −2.29627 | −2.75878 | 3 | 3 | 3 | 10.6 |
|  | hydrolase |  |  |  |  |  |  |  |  |  |
|  | Leucine-rich repeat and |  |  |  |  |  |  |  |  |  |
| Q8BLY3 | fibronectin type-III domain- | Lrfn3 | −3.63009 | −2.65855 | −1.98999 | −2.15807 | 8 | 8 | 8 | 18.2 |
|  | containing protein 3 |  |  |  |  |  |  |  |  |  |
| Q542U7;P62313 | U6 snRNA-associated Sm-like protein LSm6 | Lsm6 | 2.31678 | 2.13305 | 1.97052 | 2.04571 | 3 | 3 | 3 | 36.2 |
| P51885 | Lumican | Lum | −4.26985 | −2.13147 | −3.00873 | −5.2169 | 2 | 2 | 1 | 5.9 |
| Q60700 | Mitogen-activated protein kinase kinase kinase 12 | Map3k12 | −2.70387 | −5.98862 | −3.45255 | −2.23092 | 6 | 6 | 6 | 10.6 |
| Q14BB9 | MAP6 domain-containing protein 1 | Map6d1 | −2.05509 | −1.42188 | −1.15867 | 1.45765 | 6 | 6 | 6 | 59.2 |
| O88735 | Ensconsin | Map7 | −4.60605 | −5.50743 | −4.89465 | −4.69549 | 11 | 1 | 1 | 20 |
| Q5U421;P47811-  4;P47811-  3;P47811;B2KF34;B2KF3  5;P47811-2 | Mitogen-activated protein kinase;Mitogen-activated protein kinase 14 | Mapk14 | −2.12647 | −1.1122 | −3.40571 | −1.34655 | 5 | 5 | 5 | 16.7 |
| A2JY28;O08644;O08644-  3;O08644-2 | Ephrin type-B receptor 6 | Mep;Ephb6 | −6.2007 | −5.23567 | −4.29319 | −4.37898 | 4 | 4 | 4 | 6.6 |
| Q91YR5;Q3TRF3;Q91YR5  -1 | Methyltransferase-like protein  13 | Mettl13 | −1.93595 | −0.93314 | −2.42179 | −1.27971 | 8 | 8 | 8 | 14.2 |
| Q61733 | 28S ribosomal protein S31, mitochondrial | Mrps31 | −3.71762 | −3.78015 | −3.06893 | −2.96379 | 6 | 6 | 6 | 15.6 |
| G5E889;Q923Z3 | Protein MTO1 homolog, mitochondrial | Mto1 | −6.8225 | −2.78163 | −3.44746 | −2.41021 | 6 | 6 | 6 | 13.3 |
| B2RY26;B2RXX9;Q91Z83 | Myosin-7 | Myh7 | −1.8606 | −5.25566 | −7.55822 | −5.58141 | 26 | 6 | 2 | 12.9 |
| Q5SWP3 | NAC-alpha domain-containing protein 1 | Nacad | −2.50077 | −2.80569 | −1.26738 | −1.59517 | 15 | 15 | 15 | 16.2 |
| Q7TMW6;Q7TMW6-2 | Cytosolic Fe-S cluster assembly factor NARFL | Narfl | −4.53412 | −3.88363 | −2.30856 | −1.8654 | 7 | 7 | 7 | 19.1 |
| Q9ERR1-2;Q9ERR1 | Nuclear distribution protein nudE-like 1 | Ndel1 | −5.23318 | −0.587963 | 0.185613 | 0.168113 | 5 | 5 | 3 | 15.6 |
| F6XCS1;A2AI05-  2;A2AI05;A2AI05-3 | NADPH-dependent diflavin oxidoreductase 1 | Ndor1 | −6.03214 | −5.55681 | −1.23088 | −2.14896 | 2 | 2 | 2 | 11.1 |
| Q69ZP4;Q8C4Y3 | Negative elongation factor B | Nelfb | −0.54148 | −2.21529 | −4.50209 | −4.31543 | 7 | 7 | 7 | 18.8 |
| B2RXQ6;Q8BNJ6;Q8BNJ  6-2 | Neuropilin and tolloid-like protein 2 | Neto2 | −5.79234 | −4.93599 | −4.53387 | −4.08881 | 4 | 4 | 4 | 9.3 |
| Q9DB96 | Neuroguidin | Ngdn | −4.32016 | −5.08137 | −4.15973 | −4.55142 | 1 | 1 | 1 | 4.4 |
| Q3TFI4;Q9CQM0 | Nicolin-1 | Nicn1 | −7.14226 | −5.18235 | −1.29414 | −0.825019 | 1 | 1 | 1 | 6.6 |
| B0F2B4 | Neuroligin 4-like | Nlgn4l | −2.81178 | −2.43228 | −2.39803 | −2.7964 | 10 | 7 | 7 | 16.2 |
| Q8R5K4-  2;Q8R5K4;Q3U5C6 | Nucleolar protein 6 | Nol6 | −2.26949 | −4.04373 | −5.94562 | −5.97052 | 3 | 3 | 3 | 3.3 |

| E9PUN2;Q3TQ54;E9PUN  1 | N-terminal Xaa-Pro-Lys N- methyltransferase 1;N-terminal | Nrxn2 | −1.25204 | −1.47424 | −2.4178 | −2.34696 | 10 | 3 | 3 | 19.2 |
| --- | --- | --- | --- | --- | --- | --- | --- | --- | --- | --- |
| Q8R2U4;A2APZ3 | Xaa-Pro-Lys N-methyltransferase 1, N- | Ntmt1 | −1.84659 | −0.842786 | −2.01886 | −2.23928 | 3 | 3 | 3 | 14.3 |
|  | terminally processed |  |  |  |  |  |  |  |  |  |
| E0CYQ2;Q9CQ48 | NudC domain-containing protein 2 | Nudcd2 | −0.982296 | −1.15859 | 0.661233 | 0.737509 | 2 | 2 | 2 | 22 |
| Q9CWD3;Q9CWD3-2 | Nucleoside diphosphate-linked moiety X motif 17 | Nudt17 | −1.53852 | −0.610752 | −2.37696 | −1.67712 | 2 | 2 | 2 | 11.8 |
| A8Y5T6;Q8CFI5;Q3U4M2 | Probable proline-tRNA ligase, mitochondrial | Pars2 | −1.59363 | −1.4451 | −2.71185 | −4.58667 | 9 | 9 | 9 | 25.4 |
| E9Q7L3;D3Z1W6;E9Q7L2  ;D3Z1N4;E9Q4Y5;F8VQD  1;Q8BSQ9;Q8BSQ9-  2;D3YYF2;F6THL5;D6RIL | Protein polybromo-1 | Pbrm1 | −4.26516 | −4.87931 | −6.3552 | −4.7535 | 9 | 9 | 9 | 6.1 |
| 0;D6RI94 | Pre-B-cell leukemia |  |  |  |  |  |  |  |  |  |
| Q3TVI8 | transcription factor-interacting | Pbxip1 | −1.85764 | −1.95283 | −1.6448 | −2.68755 | 4 | 4 | 4 | 7.7 |
|  | protein 1 |  |  |  |  |  |  |  |  |  |
| Q8CFQ6 |  | Pcbp3 | −5.25863 | −3.33737 | −2.38875 | −2.10359 | 11 | 1 | 1 | 59.4 |
| Q3KQH9;Q3UCC5;F8WI8  8;Q6P8I4;Q3TM89;D3YW | PEST proteolytic signal-containing nuclear protein | Pcnp | 0.508995 | −0.0084042 | 1.41107 | 1.20784 | 4 | 4 | 4 | 24.4 |
| I6L9D3;^A^Q^4^8K0D0-  2;Q8K0D0 | Cyclin-dependent kinase 17 | Pctk2;Cdk17 | −3.05312 | −1.55193 | −4.80834 | −4.11622 | 10 | 4 | 4 | 28.1 |
| Q6NS46 | Protein RRP5 homolog | Pdcd11 | −4.31508 | −5.52755 | −7.31059 | −6.10466 | 10 | 10 | 10 | 6.2 |
| Q8BVF2 | Phosducin-like protein 3 | Pdcl3 | −4.20483 | −3.53457 | −2.20458 | −2.3076 | 3 | 3 | 3 | 13.3 |
| Q3THL5;Q80X73 | Protein pelota homolog | Pelo | −0.8349 | −1.39262 | −1.91461 | −1.9417 | 7 | 7 | 7 | 19.2 |
| E9Q6U4;Q3UWL8;E9Q4Q  8;E9Q8R1;Q6P0X1 | Prefoldin subunit 4 | Pfdn4 | −0.0023344 | 0.2246 | 1.15794 | 0.873294 | 2 | 2 | 2 | 20.8 |
|  | Membrane-associated |  |  |  |  |  |  |  |  |  |
| Q3UHE1-2;Q3UHE1 | phosphatidylinositol transfer | Pitpnm3 | −0.649257 | −1.06664 | −0.348468 | −0.239872 | 16 | 16 | 16 | 25.6 |
|  | protein 3 |  |  |  |  |  |  |  |  |  |
| A2A5K2;Q4FJT5;Q3UFS5;  P55065;Q3UE59 | Phospholipid transfer protein | Pltp | −3.12222 | −1.71699 | −4.55196 | −3.49301 | 4 | 4 | 4 | 12.9 |
| Q3TYC1;Q9QYK9;Q3UYI0  ;A2ALM3 | Calcium/calmodulin-dependent protein kinase type 1B | Pnck | 2.47158 | 1.88014 | 1.74659 | 0.853406 | 12 | 12 | 12 | 55.1 |
| Q6PAM0;D6RG50 | 5-AMP-activated protein kinase subunit beta-2 | Prkab2 | −1.44987 | −0.826796 | 0.16315 | 0.641301 | 9 | 9 | 8 | 51.3 |
| Q9QYT9;Q4FJQ7;Q3TZI7;  P04925;Q3UG89;Q3UF68; | Major prion protein | Prnpb;Prnp | 1.29658 | 0.967623 | 2.91704 | 1.67065 | 5 | 5 | 5 | 31.1 |
| Q3UBH0 |  |  |  |  |  |  |  |  |  |  |
| Q9R1C7-  2;Q9R1C7;A0PJI0 | Pre-mRNA-processing factor  40 homolog A | Prpf40a | −0.567609 | −0.579863 | −2.76617 | −3.86227 | 5 | 5 | 5 | 7.9 |
| Q52KR3;Q52KR3-  6;F8QPD5;Q52KR3- | Protein prune homolog 2 | Prune2 | −4.16256 | −3.70917 | −5.56415 | −4.7021 | 8 | 8 | 8 | 3.6 |
| 3;Q52KR3-2;Q52KR3-4 |  |  |  |  |  |  |  |  |  |  |
| A0A068BIT8;P28063;G3U  ZW8 | Proteasome subunit beta type;Proteasome subunit beta type-8 | Psmb8 | −1.23564 | −1.45445 | −1.91328 | −2.32515 | 2 | 2 | 2 | 8.3 |
| Q5SSW2 | Proteasome activator complex subunit 4 | Psme4 | −3.82641 | −3.92777 | −7.29279 | −6.156 | 7 | 7 | 7 | 4.2 |
| Q3UZM8;E9PXR4;Q0PD1  1;Q8VHQ4;E9Q016 | Ras-related protein Rab-40C | Rab40c | −6.59567 | −5.20919 | −5.01176 | −3.0972 | 2 | 2 | 2 | 10.4 |
| Q61550;Q3TG35;A1L367;  A1L366;Q3UTE4 | Double-strand-break repair protein rad21 homolog | Rad21 | −2.10958 | −2.93769 | −5.30418 | −4.65189 | 10 | 10 | 10 | 22.7 |
| Q3U405;P58069;D3YZE9 | Ras GTPase-activating protein  2 | Rasa2 | −4.47621 | −3.76671 | −5.18442 | −5.9755 | 7 | 6 | 6 | 10 |
| Q80YQ8 | Protein RMD5 homolog A | Rmnd5a | −2.0936 | −1.75808 | −3.05393 | −2.61592 | 3 | 3 | 3 | 8.7 |
| Q99KL9;Q3TE40;Q62193;  F6V8R7 | Replication protein A 32-kDa subunit | Rpa2 | −2.34437 | −2.14662 | −1.85082 | 0.185583 | 3 | 3 | 3 | 12.6 |
| Q80TE0;Q80TE0-  2;V9GWX3 | RNA polymerase II-associated protein 1 | Rpap1 | −4.94054 | −5.10154 | −3.61129 | −2.90548 | 12 | 12 | 12 | 12.2 |
| G3X926;Q9JJ80;S4R2H5;  S4R1Y5;S4R2R2 | Ribosome production factor 2 homolog | Rpf2 | −4.02907 | −4.69339 | 0.0986386 | −3.62292 | 5 | 5 | 5 | 22.7 |
| Q9Z2B9 | Ribosomal protein S6 kinase alpha-4 | Rps6ka4 | −4.97595 | −4.89324 | −5.73199 | −6.25624 | 5 | 4 | 4 | 7.5 |
| Q6IM74;Q6IM75;O70622-  2;O70622;Q3TUZ6 | Reticulon;Reticulon-2 | Rtn2 | 0.143135 | −0.0189791 | −0.999139 | −0.177105 | 3 | 3 | 3 | 27 |
| A2ATS6;Q7M6Z0 | Reticulon-4 receptor-like 2 | Rtn4rl2 | −1.60449 | −1.56087 | −6.08083 | −3.32554 | 6 | 6 | 6 | 16 |
| G5E8A7;Q80XS6 | Protein Smaug homolog 2 | Samd4b | −3.07537 | −3.45181 | −5.20379 | −6.36674 | 4 | 4 | 4 | 6.3 |
| Q6DID3;Q3TZC7 | Protein SCAF8 | Scaf8 | −2.76494 | −2.86536 | −4.53414 | −4.18599 | 4 | 4 | 2 | 3.9 |
| E9PW82;B2RY06;B1AYL0 | Sodium channel |  |  |  |  |  |  |  |  |  |
| ;B7ZWN0;Q62205;F8VPQ | protein;Sodium channel protein | Scn9a | −0.716043 | −2.23694 | −5.50036 | −5.87552 | 22 | 11 | 10 | 13.1 |
| 7 | type 9 subunit alpha |  |  |  |  |  |  |  |  |  |
| Q00898 | Alpha-1-antitrypsin 1-5 | Serpina1e | −5.55248 | −6.44106 | −1.76656 | −1.38584 | 6 | 2 | 2 | 16.5 |
| Q8VHL1 | Histone-lysine N- methyltransferase SETD7 | Setd7 | −1.07561 | −1.17324 | −0.0276107 | 1.14211 | 7 | 7 | 7 | 33.3 |
| E9QQ33;Q69ZI1-  2;Q69ZI1;Q69ZI1-  3;Q69ZI1-4 | E3 ubiquitin-protein ligase SH3RF1 | Sh3rf1 | −4.87125 | −4.87688 | −3.15888 | −2.51028 | 6 | 6 | 6 | 11.4 |
| Q3URY3 |  | Shc3 | −5.84198 | −3.33601 | −3.11307 | −4.25087 | 3 | 1 | 1 | 22.5 |
| Q8CI08;Q8CI08-2 | SLAIN motif-containing protein  2 | Slain2 | −6.69511 | −6.80623 | −2.43381 | −3.67066 | 3 | 3 | 3 | 7.4 |

| D3YVC9;Q8C0K5 | Graves disease carrier protein homolog | Slc25a16 | −2.98655 | −2.55855 | −5.59476 | −4.61202 | 3 | 3 | 3 | 9.3 |
| --- | --- | --- | --- | --- | --- | --- | --- | --- | --- | --- |
| O35488;A2ANX6;Q3UNR3 | Very long-chain acyl-CoA | Slc27a2 | −2.02441 | −1.79984 | −1.52565 | −1.01281 | 8 | 8 | 8 | 17.7 |
| ;Q3TN99 | synthetase |  |  |  |  |  |  |  |  |  |
| Q3TQZ8;E9Q3N1;Q3UTL4 | High affinity cationic amino | Slc7a1 | −2.94001 | −1.60696 | −3.6764 | −2.89026 | 2 | 1 | 1 | 6.7 |
| ;Q3UGD6;Q09143 | acid transporter 1 |  |  |  |  |  |  |  |  |  |
| P61406;F6WT10 | Telomerase-binding protein EST1A | Smg6 | −6.52359 | −2.27224 | −5.69585 | −4.89234 | 4 | 4 | 4 | 3.9 |
| P70158 | Acid sphingomyelinase-like | Smpdl3a | −6.23765 | −4.64273 | −2.60532 | −3.45199 | 4 | 4 | 4 | 12.4 |
|  | phosphodiesterase 3a |  |  |  |  |  |  |  |  |  |
| E9PVQ3;Q91WJ7-  2;Q91WJ7;E9Q7S4;E9PZ | SPATS2-like protein | Spats2l | −5.22955 | −5.8709 | −4.72531 | −3.51086 | 4 | 4 | 4 | 10.9 |
| E9;E9Q106 |  |  |  |  |  |  |  |  |  |  |
| E9PUC4;Q6NSW3- | A-kinase anchor protein SPHKAP |  |  |  |  |  |  |  |  |  |
| 2;Q6NSW3- |  | Sphkap | −5.55286 | −4.99565 | −4.2598 | −4.38318 | 8 | 8 | 8 | 7.1 |
| 4;Q6NSW3;Q6NSW3-3 |  |  |  |  |  |  |  |  |  |  |
| F8WGW3;Q497V5-  2;Q497V5;B7ZWI4;Q8CH | S1 RNA-binding domain- | Srbd1 | −7.09229 | −6.67808 | −6.18014 | −4.83515 | 4 | 4 | 4 | 5.4 |
| Z0 | containing protein 1 |  |  |  |  |  |  |  |  |  |
| P54116;Q99L68;Q8BPA1 | Erythrocyte band 7 integral | Stom | −4.2501 | −1.97073 | −3.81604 | −5.26844 | 3 | 3 | 3 | 13.7 |
|  | membrane protein |  |  |  |  |  |  |  |  |  |
| Q9Z2P1;Q6PGE2;Q8BNE |  | SUR-1;Abcc8 | −4.10095 | −4.0335 | −6.56303 | −4.67266 | 5 | 5 | 5 | 7.9 |
| 2;B2RUS7 |  |  |  |  |  |  |  |  |  |  |
| E3VVQ8;F6R6A4;Q7TMJ2  ;E9Q983;Q8K4L2;E9Q3Z5 | Supervillin | Svil | −5.49601 | −5.57157 | −4.11465 | −3.15186 | 4 | 4 | 4 | 2.7 |
| ;Q8K4L3 |  |  |  |  |  |  |  |  |  |  |
| Q9CY49;Q9R099 | Transducin beta-like protein 2 | Tbl2 | −1.19382 | −0.0188527 | −1.00201 | −1.46433 | 7 | 7 | 7 | 18.6 |
| Q8C4J7;Q8CE86;A0JLN6 | Transducin beta-like protein 3 | Tbl3 | −4.27123 | −3.75093 | −5.15721 | −5.82649 | 6 | 6 | 6 | 12.2 |
| Q9EPQ8- |  |  |  |  |  |  |  |  |  |  |
| 2;Q9EPQ8;Q66JM6;Q80U  46 | Transcription factor 20 | Tcf20 | −5.5664 | −7.60396 | −2.44306 | −4.78839 | 7 | 7 | 7 | 6 |
| Q91W18-2;Q91W18- | Tudor domain-containing | Tdrd3 | −3.85237 | −4.66564 | −4.69924 | −4.58293 | 3 | 3 | 3 | 5.4 |
| 3;Q91W18;E9Q6T6 | protein 3 |  |  |  |  |  |  |  |  |  |
| Q921I1 | Serotransferrin | Tf | 0.97093 | 0.798816 | −0.0840255 | −0.547178 | 30 | 3 | 3 | 51.9 |
| Q80TG4;F6ZBY9;Q3UY41  ;Q62441;Q6PFG2;Q5SQA | Transducin-like enhancer |  |  |  |  |  |  |  |  |  |
| 2;E9Q2Q6;Q5SQA4;Q5S | protein 4;Transducin-like | Tle4;Tle1 | −5.16952 | −4.23386 | −2.80857 | −2.96057 | 6 | 6 | 3 | 10.3 |
| QA3;Q62440-3;Q62440-  2;Q62440- | enhancer protein 1 |  |  |  |  |  |  |  |  |  |
| 5;Q62440;Q62440-6 |  |  |  |  |  |  |  |  |  |  |
| E0CZE8;E0CXR0;E0CX98 | Bax inhibitor 1 | Tmbim6 | 0.548367 | 0.974124 | 0.902712 | 0.739553 | 2 | 2 | 2 | 65.6 |
| ;Q9D2C7 |  |  |  |  |  |  |  |  |  |  |
| Q9CQE2 | Transmembrane protein 223 | Tmem223 | −1.78208 | −2.39038 | −3.35743 | −5.35721 | 3 | 3 | 3 | 17.6 |
| Q8BHW5- |  |  |  |  |  |  |  |  |  |  |
| 3;Q8BHW5;Q8BHS0;Q8B | Transmembrane protein 255A | Tmem255a | −2.85063 | −2.9795 | −1.81424 | −1.59866 | 2 | 2 | 2 | 6 |
| HW5-2 |  |  |  |  |  |  |  |  |  |  |
| Q9JJG6;Q9JJG6-2 | Transmembrane protein 47 | Tmem47 | 2.40143 | 2.14212 | 2.40106 | 1.67779 | 2 | 2 | 2 | 8.8 |
| Q8QZR0 | Translocating chain-associated | Tram1l1 | −1.34948 | −1.15557 | −5.55519 | −5.80247 | 1 | 1 | 1 | 3.3 |
|  | membrane protein 1-like 1 |  |  |  |  |  |  |  |  |  |
| A2RSY6-2;A2RSY6 | TRMT1-like protein | Trmt1l | −7.00995 | −5.46259 | −3.98527 | −5.09783 | 3 | 3 | 3 | 6.3 |
| Q9DAT5 | Mitochondrial tRNA-specific 2- thiouridylase 1 | Trmu | −0.788825 | −0.216366 | −1.01088 | −0.922755 | 12 | 12 | 9 | 41 |
| Q4VA41;P50586- | Tubby-like protein;Tubby | Tub | −0.735819 | −0.238284 | −1.35545 | −0.402042 | 10 | 10 | 10 | 26.9 |
| 2;P50586 | protein |  |  |  |  |  |  |  |  |  |
| Q542N4;Q9WVF8 | Tumor suppressor candidate 2 | Tusc2 | −0.350006 | 0.152354 | 1.77005 | 1.77048 | 2 | 2 | 2 | 30.9 |
| F6SPK0;Q3TV38;Q9JJZ4;  Q3UDP8 | Ubiquitin-conjugating enzyme  E2 J1 | Ube2j1 | −2.01138 | −1.69972 | −1.33276 | 0.165133 | 7 | 7 | 7 | 36.6 |
| Q78JW9 | Ubiquitin domain-containing protein UBFD1 | Ubfd1 | 3.96021 | 2.38592 | 4.54429 | 6.13487 | 8 | 8 | 8 | 23.4 |
| E9Q2H1;Q80TP3 | E3 ubiquitin-protein ligase UBR5 | Ubr5 | −3.88164 | −7.61186 | −7.67911 | −5.09796 | 6 | 6 | 5 | 3.4 |
| Q3ULL6;Q9CS15;Q80UI8 | Uracil | Upf3b | −0.256068 | −0.378957 | −2.42019 | −3.31287 | 9 | 9 | 9 | 22.5 |
| B1AVZ0 | phosphoribosyltransferase | Uprt | −0.382964 | −1.52771 | −0.116226 | 0.0728529 | 6 | 6 | 6 | 26.8 |
|  | homolog |  |  |  |  |  |  |  |  |  |
|  | Ubiquinol-cytochrome-c |  |  |  |  |  |  |  |  |  |
| D3Z4C9;Q9CQY6;D3Z4D6 | reductase complex assembly | Uqcc2 | 2.2288 | 2.08884 | 0.861479 | 0.96457 | 4 | 4 | 4 | 37 |
|  | factor 2 |  |  |  |  |  |  |  |  |  |
| P99028 | Cytochrome b-c1 complex | Uqcrh | 2.28145 | 2.46744 | 3.07365 | 3.37063 | 3 | 3 | 3 | 47.2 |
|  | subunit 6, mitochondrial Probable U3 small nucleolar RNA-associated protein 11 |  | −3.48055 | −5.40431 | −6.21635 | −6.01341 | 2 | 2 | 2 | 14.7 |
| Q9D948;Q9CZJ1 |  | Utp11l |  |  |  |  |  |  |  |  |
| Q6ZPV8;Q91ZD4;D3YY75 | Vang-like protein 2 | Vangl2 | −1.68588 | −1.42599 | −2.60873 | −1.70034 | 7 | 7 | 7 | 13.7 |
| Q80TR8-3;Q80TR8-  4;Q80TR8;Q80TR8-2 | Protein VPRBP | Vprbp | −3.92781 | −5.14603 | −6.33703 | −5.44521 | 6 | 6 | 6 | 8.1 |
| Q3T9Z7;Q8BH43;Q80UQ8 | Wiskott-Aldrich syndrome protein family member 2 | Wasf2 | −1.73342 | −1.34717 | −3.111 | −3.48158 | 5 | 5 | 5 | 13.1 |
| Q4V9X1;Q9JJA4;D3Z369 | Ribosome biogenesis protein WDR12 | Wdr12 | −3.49791 | −2.97507 | −2.29346 | −0.466236 | 3 | 3 | 3 | 9.2 |
| K4DI77;Q5ND34- | WD repeat-containing protein | Wdr81 | −3.15013 | −4.59589 | −2.16922 | −1.92129 | 14 | 14 | 14 | 10.1 |
| 2;Q5ND34;Q5ND34-3 | 81 |  |  |  |  |  |  |  |  |  |
| Q0KIX1;Q5SXA9 | Protein KIBRA | Wwc1 | −5.00956 | −5.28783 | −3.88823 | −4.19935 | 4 | 4 | 4 | 5.9 |
| Q6NXJ0 | Protein WWC2 | Wwc2 | 1.19592 | 0.339178 | 0.738812 | −1.46903 | 4 | 4 | 4 | 5.1 |
| P23475;E9PZD4 | X-ray repair cross­complementing protein 6 | Xrcc6 | −2.89602 | −4.48912 | −5.81669 | −5.50742 | 5 | 5 | 5 | 8.2 |
| Q0VFY6;Q80TN5- | Palmitoyltransferase;Palmitoyltr | Zdhhc17 | −2.63491 | −2.78704 | −1.28554 | −0.954442 | 5 | 5 | 5 | 11.6 |
| 2;Q80TN5;Q3TRB3 | ansferase ZDHHC17 |  |  |  |  |  |  |  |  |  |
| Q3U7F6;Q9ERU3 | Zinc finger protein 22 | Zfp422;Znf22 | −2.03756 | −0.913177 | −1.88367 | −1.96788 | 3 | 3 | 3 | 15.6 |

| H3BL53;G3UYC1;G3UY05  ;Q8C6P8-2;Q8C6P8 | Zinc finger protein 57 | Zfp57 | 0.8329 | 3.88376 | 0.347972 | 0.611119 | 1 | 1 | 1 | 6.8 |
| --- | --- | --- | --- | --- | --- | --- | --- | --- | --- | --- |
| Q6ZPK7 | Lateral signaling target protein  2 homolog | Zfyve28 | −4.65797 | −4.41992 | −5.69848 | −4.64481 | 1 | 1 | 1 | 1.7 |
| Q9CRC6 | UPF0693 protein C10orf32 |  | −6.32359 | −6.40211 | −1.61219 | −1.11151 | 3 | 3 | 3 | 44.8 |
|  | homolog |  |  |  |  |  |  |  |  |  |
